# WOX1 controls leaf serration development via temporally restricting BRASSINAZOLE RESISTANT 1 and CUP SHAPED COTYLEDON 3 expression in Arabidopsis

**DOI:** 10.1093/jxb/erae443

**Published:** 2024-10-30

**Authors:** Lian Xu, Nimeng Fang, Ting Lu, Toshiaki Tameshige, Miyuki T Nakata, Yuli Jiang, Li Tan, Hai He, Xuelin Zhang, Yimei Huang, Caiming Li, Zhenbiao Yang, Wenxin Tang, Shingo Nagawa

**Affiliations:** College of Life Sciences, Fujian Agriculture and Forestry University, Fuzhou, Fujian, China; Horticultural Biology and Metabolomics Center, Haixia Institute of Science and Technology, Fujian Agriculture and Forestry University, Fuzhou, Fujian, China; College of Life Sciences, Fujian Agriculture and Forestry University, Fuzhou, Fujian, China; Horticultural Biology and Metabolomics Center, Haixia Institute of Science and Technology, Fujian Agriculture and Forestry University, Fuzhou, Fujian, China; College of Life Sciences, Fujian Agriculture and Forestry University, Fuzhou, Fujian, China; Plant Synthetic Biology Center, Haixia Institute of Science and Technology, Fujian Agriculture and Forestry University, Fuzhou, Fujian, China; Kihara Institute for Biological Research (KIBR), Yokohama City University, 641-12 Maioka, Totsuka-ward, Yokohama 244-0813, Japan; Division of Biological Science, Graduate School of Science and Technology, Nara Institute of Science and Technology (NAIST), 8916-5 Takayama-Cho, Ikoma, Nara 630-0192, Japan; Graduate School of Life and Environmental Sciences, Kyoto Prefectural University, Kyoto 606-8522, Japan; Division of Biological Science, Graduate School of Science and Technology, Nara Institute of Science and Technology (NAIST), 8916-5 Takayama-Cho, Ikoma, Nara 630-0192, Japan; Center for Digital Green-innovation, Nara Institute of Science and Technology (NAIST), 8916-5 Takayama-Cho, Ikoma, Nara 630-0192, Japan; Faculty of Advanced Science and Technology, Kumamoto University, 2-39-1 Kurokami, Chuo-ku, Kumamoto 860-8555Japan; Shanghai Center for Plant Stress Biology, CAS Center for Excellence in Molecular Plant Sciences, Chinese Academy of Sciences (CAS), Shanghai 201602China; University of Chinese Academy of Sciences, 100049 Beijing, China; Shanghai Center for Plant Stress Biology, CAS Center for Excellence in Molecular Plant Sciences, Chinese Academy of Sciences (CAS), Shanghai 201602China; Horticultural Biology and Metabolomics Center, Haixia Institute of Science and Technology, Fujian Agriculture and Forestry University, Fuzhou, Fujian, China; College of Life Sciences, Fujian Agriculture and Forestry University, Fuzhou, Fujian, China; Horticultural Biology and Metabolomics Center, Haixia Institute of Science and Technology, Fujian Agriculture and Forestry University, Fuzhou, Fujian, China; Horticultural Biology and Metabolomics Center, Haixia Institute of Science and Technology, Fujian Agriculture and Forestry University, Fuzhou, Fujian, China; College of Horticulture, China Agricultural University, Beijing 100069, China; College of Life Sciences, Fujian Agriculture and Forestry University, Fuzhou, Fujian, China; Horticultural Biology and Metabolomics Center, Haixia Institute of Science and Technology, Fujian Agriculture and Forestry University, Fuzhou, Fujian, China; Horticultural Biology and Metabolomics Center, Haixia Institute of Science and Technology, Fujian Agriculture and Forestry University, Fuzhou, Fujian, China; Plant Synthetic Biology Center, Haixia Institute of Science and Technology, Fujian Agriculture and Forestry University, Fuzhou, Fujian, China; Horticultural Biology and Metabolomics Center, Haixia Institute of Science and Technology, Fujian Agriculture and Forestry University, Fuzhou, Fujian, China; University of Sydney, Australia

**Keywords:** Arabidopsis, BZR1, CUC3, tooth, WOX1

## Abstract

Leaves have evolved shape diversity, ranging from simple leaves with a smooth margin to complicated shapes with toothed/serrated, lobed, and dissected leaves with leaflets. In the model plant *Arabidopsis thaliana* with simple leaves producing a serrated margin, boundary regulatory factor genes *CUP SHAPED COTYLEDON* 2 (*CUC2*) and *CUC3* play important roles in promoting leaf initiation and maintenance of serration. Stem cell-related *WUSCHEL-RELATED HOMEOBOX1* (*WOX1*) and *PRESSED FLOWER* (*PRS*)/*WOX3* are also essential for leaf margin morphogenesis, but the role of WOX1 and PRS as well as the relationships between CUCs and WOXs for tooth development are unclear. In this study, we found that WOX1, but not PRS, prevents overproduction of number of teeth and excessive tooth size by limiting *CUC3* expression to a moderate level in a temporally regulated manner. We also revealed that BRASSINAZOLE RESISTANT 1 (BZR1), a known regulator of plant development including boundary regions, is involved in WOX1 negative regulation of tooth development by repressing *CUC3* expression during the initiation/early stage of tooth development. WOX1 parallelly limits *BZR1* and *CUC3* expression from the late stage of the first two teeth, while it restricts CUC3 activity in a BZR1-dependent manner from the initiation/early stage of subsequently developed teeth. This study uncovers a new mechanism for WOX1 in fine-tuning the leaf margin geometry.

## Teaser

WOX1, a stem cell factor, plays vital roles in balancing leaf tooth outgrowth extent and number via restricting *CUC3* and *BZR1* expression to a moderate level in a temporally regulated manner.

## Introduction

Leaves exhibit a remarkable diversity in shape, with their distinctiveness primarily determined by marginal morphological diversity. This ranges from simple leaves, either with or without marginal serrations known as leaf teeth, to highly dissected leaves with leaflets. These diverse leaf morphologies contribute significantly to plant environmental adaptations. For instance, leaves with serrated edges appear to enable plants to adapt to cold climates (Royer and Wilf, 2006; Peppe *et al*., 2011). In the model plant *Arabidopsis*, leaves are simple with variable degrees of serrations. Young leaves initially have smooth margins, but as they grow to ~250 μm, the first pair of leaf teeth emerges at the margin (Nikovics *et al*., 2006). These teeth gradually enlarge and increase in number as the leaves continue to develop.

Organ boundaries play important roles in organogenesis in plants and animals. They are domains with restricted cell growth governed by boundary genes, and are required to delimitate cell territories between different cell types separating adjacent organs/tissues (Zadnikova and Simon, 2014). In leaves with serrated margins, the boundary region referred to as the sinus is crucial for separating the successively emerging teeth. The boundary *CUP SHAPED COTYLEDON* (*CUC*) genes play vital roles in defining the organ boundary in diverse developmental contexts in the aerial parts of plants, including leaf margin morphogenesis (Aida *et al*., 1997; Nikovics *et al*., 2006; Hasson *et al*., 2011; Gendron *et al*., 2012; Galbiati *et al*., 2013; Hepworth and Pautot, 2015). Both *CUC2* and *CUC3* are plant-specific *NAC* transcription factor genes that exhibit partially overlapping expression patterns in developing leaf teeth. *CUC2* exhibits a broader expression domain encompassing the sinus region, while *CUC3* expression is confined to a few cells within the sinus *CUC2* expression domain (Nikovics *et al*., 2006; Hasson *et al*., 2011; Serra and Perrot-Rechenmann, 2020). CUC2 is primarily distributed along the leaf margin during the emerging phase of leaf primordia to promote the initiation of serration, and is gradually restricted to the flanking region between serrations to inhibit cell growth to sustain growth of the protrusion (Nikovics *et al*., 2006; Bilsborough *et al*., 2011; Kierzkowski *et al*., 2019). *CUC3* has also been reported to be involved in regulating leaf shape (Hasson *et al*., 2011). It functions as a local downstream relay of *CUC2* to promote leaf tooth outgrowth early on via activating a strong auxin response at the tooth tip and inhibiting cell growth at the sinus (Maugarny-Cales *et al*., 2019; Serra and Perrot-Rechenmann, 2020). Although CUC2 and CUC3 are functionally redundant as a pathway, they are differentially regulated by several factors. In *Arabidopsis* leaves, *MIR164A* reduces the expression of its target *CUC2*, but not *CUC3*, to result in a moderate output level of CUC2 activity in order to maintain the proper extent of growth of leaf serrations (Rhoades *et al*., 2002; Nikovics *et al*., 2006). Whether another mechanism exists in balancing serration growth extent/number through differential regulation of CUCs remains to be investigated.

Brassinosteroid (BR) is a growth-promoting hormone that regulates a diverse array of developmental processes including the development of ovule primordia, shoot regeneration, and the formation of organ boundaries (Cheon *et al*., 2010; Gendron *et al*., 2012; Huang *et al*., 2013). The suppression of BR signaling is crucial for the appropriate establishment of organ boundaries within the shoot apical meristem. Excessive accumulation of BZR1, a transcription factor involved in BR-mediated signaling, can lead to aberrant organ boundary phenotypes due to its direct binding to the CUC2 and CUC3 promoters (Gendron *et al*., 2012). Furthermore, transgenic tomato plants overexpressing BRI1 (the BR receptor) exhibit smooth leaf margins, suggesting a conserved mechanism of BR signaling in regulating morphogenesis at organ boundaries across diverse plant species (Holton *et al*., 2007).

The *WUSCHEL-RELATED HOMEOBOX* (*WOX*) genes belong to a family of homeodomain transcription factor genes that are suggested to play crucial roles in region-specific programming (Zhao *et al*., 2009, 2015; Lie *et al*., 2012; Ohmori *et al*., 2013; Zhang *et al*., 2017; Ikeuchi *et al*., 2022; Satterlee *et al*., 2023; Tanaka *et al*., 2023). In *Arabidopsis* emerging leaves, *WOX1* and *PRESSED FLOWER* (*PRS*)/*WOX3* are expressed along the leaf margin, with their expression gradually restricted to the basal region as the leaf grows (Alvarez *et al*., 2016). These genes are essential for the transitional marginal meristem activity that contributes to leaf blade outgrowth (Nakata *et al*., 2012). While ectopic expression of WOX1 has been reported to elevate the transcripts of *CUC2* and *CUC3* (Nakata *et al*., 2018), it remains unclear whether WOX1 and PRS are involved in the development of serration through the regulation of boundary regulators such as CUCs. The TEOSINTE BRANCHED1, CYCLOIDEA, and PCF (TCP) family of transcription factors has been reported to negatively regulate *WOX* gene expression during leaf margin morphogenesis (Alvarez *et al*., 2016). Moreover, TCP4 directly inhibits CUC2 and CUC3 activity during age-dependent changes of leaf complexity within a single plant (Koyama *et al*., 2007, 2017; Rubio-Somoza *et al*., 2014; Schommer *et al*., 2014). These findings suggest a potential correlation between WOXs and CUCs during leaf margin morphogenesis.

In this study, we present evidence for the key roles of WOX1 in regulating the growth and number of serrations. We find that WOX1 temporally controls *CUC3* expression to sustain CUC3 activity at a moderate level. Further investigation reveals that WOX1 prevents tooth growth via inhibiting BZR1 and CUC3 activity at the late stage of tooth development, while suppressing tooth development from the initiation/early stage via restriction of *CUC3* expression in a BZR1-dependent manner. These results show that WOX1 acts as a regulator to achieve an appropriate development of leaf serrations.

## Materials and methods

### Plant material and growth condition

All transgenic plants and mutants were generated in the Col-0 background. The *wox1-101* and *prs-2* (Nakata *et al*., 2012), *cuc3-105* (Hibara *et al*., 2006), *bzr1-1* (Saito *et al*., 2018), and *bzr1-1D* (Wang *et al*., 2002) mutants were previously described. Seeds were sown on half-strength Murashige and Skoog medium, and were stratified at 4 °C for 2 d before being transferred to a growth chamber for 7 d. The seedlings were grown on soil in a growth room at 22 °C, 60% relative humidity under long-day photoperiods (16 h/8 h light/dark).

### Plasmid construction

The *pWOX1:2GFP-WOX1* construct (Caggiano *et al*., 2017) used to complement the abnormal tooth protrusion phenotype in *wox1-101* and *wox1-101 prs-2* mutants was obtained from Dr Marcus G. Heisler. For the construction of *pCUC2:CUC2-YFP*, the 3.2 kb CUC2 promoter used in a previously described *pCUC2:CUC2-VENUS* reporter (Heisler *et al*., 2005) was amplified using primer pCUC2-F/pCUC2-R, and inserted into binary vector pGWB641 carrying yellow fluorescent protein (YFP) in place of the 35S promoter (*CUC2pro:*-GWB641). The CUC2 genomic DNA without the stop codon was amplified using primer gCUC2-F/gCUC2-R, and cloned into the expression vector *CUC2pro:*-GWB641. For the construction of *pCUC3:YFP-NLS*, the 4.3 kb CUC3 promoter was amplified using primer pCUC3-F/pCUC3-R and cloned into the binary vector pBGYN which carries YFP–nuclear localization siignal (NLS). For the *pBZR1:BZR1-YFP* construct, the same length of promoter sequence was used as previously described for the *pBZR1:BZR1-YFP* reporter (Gendron *et al*., 2012). A 1 kb segment of the 5' intergenic region with the full-length BZR1 genomic sequence without a stop codon was amplified using primer pBZR1-F/BZR1-R and cloned into pGWB641. Primer sequences are listed in Supplementary Table S1. All the binary vectors used in this study give spectinomycin resistance in bacteria and Basta resistance in plants. The floral dipping method was adopted for plant transformation using *Agrobacterium tumefaciens* strain GV3101. All mutant plants carrying transgenes or other mutant genotypes were generated by crossing and confirmed by genotyping or sequencing.

### Phenotypic analysis

Several shape parameters (tooth height, weight, tooth aspect ratio, and solidity) were used in this study. A tip and two consecutive sinuses were selected for each tooth. Tooth width is defined as the distance between the two sinuses. Tooth height is the perpendicular distance from the tooth tip to the widest segment. The tooth aspect ratio refers to tooth height/width as previously reported (Maugarny-Cales *et al*., 2019). The fifth leaves of 11- to 18-day-old plants were used for the quantification of tooth height and width, and the third leaves of 12-day-old plants were obtained for the calculation of solidity. Leaves were dissected and immersed in water between a slide and coverslip for photographing. Leaf images with a leaf blade longer than 2 mm were acquired using a stereomicroscope (Nikon SMZ18), and images with leaf length less than 2 mm were obtained using an upright microscope (Nikon ECLIPSE Ni-U). The tooth height/width and solidity were measured with Fiji software, and solidity was measured from the binary blade-only images. As solidity ranges from nearly 0 (heavily serrated) to 1 (totally smooth), ‘1–Solidity’ was adopted as the tooth growth level, as previously reported (Tameshige *et al*., 2016).

### Serration positioning

The D/L ratio, that is the distance of the distal sinus of the tooth on the leaf axis to the leaf base (D) divided by the leaf length (L), was adopted for determination of the serration position as described in Biot *et al*. (2016). The proximal–distal position of the distal sinus of teeth 1–3 relative to the leaf blade length of the fifth leaf in various ecotypes was measured.

### Confocal microscopy

All confocal imaging was performed using a Leica SP8 microscope with ×20 magnification (HC PL APO CS2, numerical aperture 0.75). Excitation of fluorescent proteins was achieved by a tunable white light laser with 488 nm for green fluorescent protein (GFP) and 514 nm for YFP and propidium iodide (PI). Images were collected using a HyD detector at 500–550 nm emission spectrum for GFP, 529–545 nm for YFP, and 600–660 nm for PI. Images in a set of experiments which need to be compared with each other were collected with the same settings.

### YFP signal intensity quantification

The quantification of the *pCUC3:YFP-NLS* signal was manually performed using ImageJ. Results are the mean intensity of the YFP signal at the tooth and/or sinus region in selected sectors containing ~90 nuclei (early stage) or 30 nuclei (late stage) in the first tooth, and ~120 nuclei in the third tooth. The *pBZR1:BZR1-YFP* signal was quantified using a similar approach. The mean intensity of the BZR1–YFP signal at the tooth and/or sinus region was also calculated using ~150 nuclei (early stage) or 40 nuclei (transition timing) in the first tooth, and ~100 nuclei in the third, while the mean intensity of the CUC2–YFP signal at the tooth and/or sinus region was quantified using ~50 nuclei (early stage) or 120 nuclei (late stage) in the first tooth. The mean intensity of the background was subtracted from the mean intensity of the YFP signal.

### Cellular measurements

For cell surface area analysis, the fifth leaves were dissected from the plants and submerged in PI solution (10 mg ml^–1^) for 5–15 min. The samples were rinsed in sterilized water three times prior to the imaging of the abaxial side of the leaf. A Leica SP8 laser scanning confocal microscope was used for imaging. *Z*-stacks were acquired using the PMT detector with a voxel resolution of 0.56 µm×0.56 µm×0.56 µm. Quantitative analysis of the cell area was performed via MorphographX (Strauss *et al*., 2022).

### Statistical analysis

Statistical analyses were performed by Student’s *t*-tests, or one-way ANOVA followed by a Turkey’s HSD (*P*<0.05). The *P*-values from the comparisons between regression lines (*y*=tooth height, *x*=leaf blade length) of two different genotypes were calculated by likelihood ratio tests using the package ‘lmtest’ version 0.9-40 of R 4.0.4 by comparing two linear models, namely ‘tooth height–leaf blade length’ and ‘tooth height–leaf blade length+genotype’.

## Results

### *wox1* shows enhanced tooth size and an increase in number of teeth compared with the wild type

**Fig. 1.**
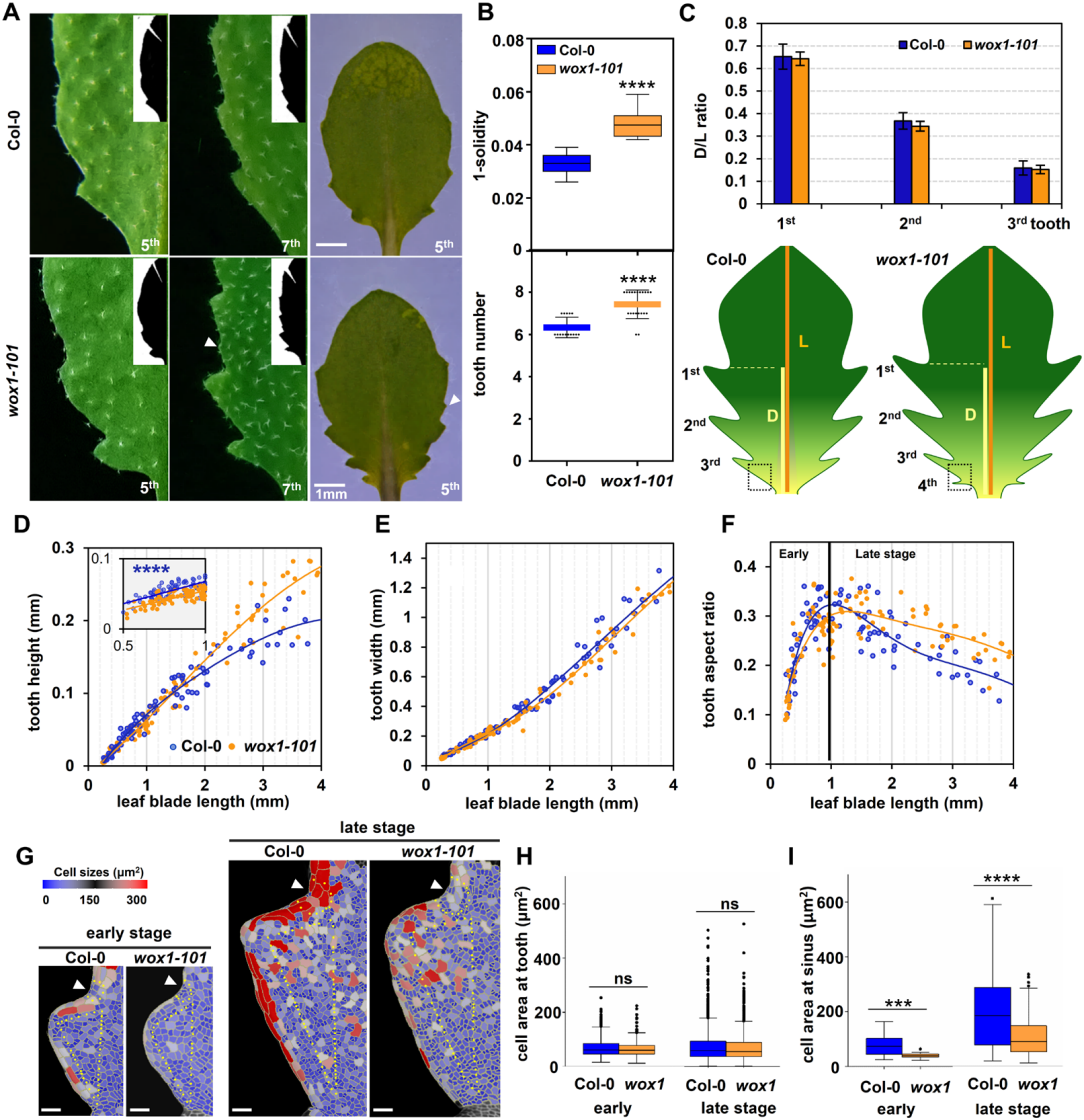
*wox1-101* shows enhanced serration size and increased tooth number. (A) Mature and/or young stage of the fifth and seventh leaves of Col-0 and *wox1-101*. Arrowheads in (A) indicate intercalary teeth, a concept put forward by Kierzkowski *et al*. (2019). (B) Tooth growth level (upper panel) and tooth number (bottom panel, only primary teeth are calculated without regard to the intercalary teeth in this study) in the fifth leaves of Col-0 (*n*=11) and *wox1-101* (*n*=12). The leaf in Col-0 possesses ~6 teeth while the leaf in *wox1-101* possesses 1–2 extra teeth compared with Col-0. Student’s *t*-test was performed for statistical analysis. ****P<0.0001. (C) Proximal–distal position of the distal sinus of teeth 1–3 relative to leaf blade length in Col-0 and *wox1-101*, as shown in Biot *et al*. (2016) (upper panel), and scheme of the fifth leaf in Col-0 and *wox1-101* (bottom panel). The black dotted square marks the placement of the fourth tooth which is inhibited in the leaf of Col-0. D, distance from the distal tooth sinus to the leaf base; L, leaf blade length from tip to the leaf base. (D–F) Quantitative analysis of tooth height (D), tooth width (E), and tooth aspect ratio (F) in the first tooth of leaf 5 in Col-0 (*n*=181) and *wox1-101* (*n*=220) correlated with elongation of leaf length. Likelihood ratio test (see the Materials and methods for details) was adopted for statistical analysis between Col-0 and *wox1-101* on tooth height at the early stage of development of the first tooth. ****P=6.31e-19. (G–I) Heatmaps of the cell area in the first tooth of leaf 5 in Col-0 and *wox1-101* at the early (~700–800 μm) and the late (~1200–1300 μm) stage points during tooth development (G). The quantitative analysis of cell area of the tooth excluding the margin cells [590 and 677 cells in Col-0 (*n*=6) and *wox1-101* (*n*=6), respectively, at the early stage; 1711 and 1464 cells in Col-0 (*n*=7) and *wox1-101* (*n*=9), respectively, at the late stage] (H) and at the sinus [55 and 67 cells in Col-0 (*n*=6) and *wox1-101* (*n*=6), respectively, at the early stage; 89 and 193 cells of Col-0 (*n*=7) and *wox1-101* (*n*=9), respectively, at the late stage] (I). White arrowheads in (G) indicate tooth sinus regions. Yellow dots in (G) at the sinus region encircle cells (including yellow dotted cells) used for cell area quantitative analysis in (I). Cells inside the yellow triangles in the tooth region in (G) are used for cell area quantitative analysis in (H). Scale bars in (G), 25 μm. Student’s *t*-test was performed for statistical analysis. ***P<0.001. ****P<0.0001. ns, non-significant.

WOX1 and PRS are expressed along the leaf margin, and the loss function of WOX1 but not PRS leads to enhanced serration size in the mature leaf (Nakata *et al*., 2012). Consistent with this, *wox1* leaves exhibit higher complexity in shape compared with Col-0 leaves (Fig. 1A, B; Supplementary Fig. S1A). Through the analysis of the number and positioning of teeth on the proximal–distal axis of the leaf blade, we found that the fifth leaf in *wox1* possesses 1–2 more teeth compared with Col-0, without affecting the placement of the first to third pair of tooth protrusions (Fig. 1C). These findings prompted us to investigate the role of WOX1 in restricting the growth of serrations.

Distinct regulatory mechanisms govern leaf margin morphology in the juvenile and mature stages (Koenig *et al*., 2009; Tameshige *et al*., 2016). To determine from which stage *wox1* exhibits serrations with enhanced size, we examined the serration developmental process in Col-0 and *wox1* during the primordial to young leaf stage. Three parameters, namely tooth width, tooth height (representing growth promotion at the protrusion tip and growth inhibition at the sinus) and the tooth aspect ratio (tooth height divided by width, reflecting anisotropic deformation during tooth growth), were analyzed, as previously described (Maugarny-Cales *et al*., 2019). In Col-0, both tooth height and width increased as the leaf grew (Fig. 1D, E; Supplementary Fig. S1B, C). A rapid increase in the tooth aspect ratio following tooth initiation was observed, until the ratio reached a peak level, and then gradually declined with leaf blade growth (Fig. 1F; Supplementary Fig. S1D).

To discriminate these two stages in our study, they were designated as the early (increase in tooth aspect ratio) and the late (decrease in tooth aspect ratio) stages, respectively. The peak level stage point serves as the demarcation point between these two phases. Despite the synchronous emergence of the teeth in *wox1* and Col-0, *wox1* exhibited reduced tooth height at the early stage but increased height at the late stage compared with Col-0 for the first two teeth (Fig. 1D; Supplementary Figs S1C, S2). The width of the first tooth in *wox1* was comparable with that in Col-0, and the second tooth exhibited a narrower width in *wox1* (Fig. 1E; Supplementary Fig. S1B). Therefore, the enhanced leaf protrusion shape in the first and second teeth of *wox1* is primarily attributed to the increased tooth height from the late rather than the early stage of tooth growth. Correspondingly, although the anisotropic tooth growth was diminished in *wox1* compared with that in the wild type at the early stage, *wox1* displayed a reduced rate of reduction of the aspect ratio at the late stage (Fig. 1F; Supplementary Fig. S1D). These abnormal phenotypes in *wox1* are recovered by the introduction of a *WOX1* genomic fragment (Supplementary Fig. S1A–F).

A further analysis of the third tooth on leaf 5 revealed that, different from the phenotype observed in the first and second teeth, *wox1* showed a greater tooth height compared with the wild type at the early stage of development (Supplementary Fig. S1G–I). All the above results suggest that WOX1 negatively regulates development of the first and second teeth from the late stage and development of the third tooth from the early stage. Consistent with the observation in the mature stage, the tooth phenotype in *prs* during the primordial to young leaf stage was also comparable with that in Col-0 at the same stage as in previous work (Nakata *et al*., 2012) (Supplementary Fig. S1A–F). These results suggest that WOX1 spatiotemporally limits tooth outgrowth as well as tooth number.

A previous report showed that local cell growth inhibition resulting in smaller cells at the sinus of the tooth margin would lead to enhanced protrusion (Hajheidari *et al*., 2019). To further elucidate the protrusion growth features that underlie the different leaf margin morphogenesis between Col-0 and *wox1*, we conducted a cell-level analysis of the first tooth of leaf 5. Two stage points in Col-0 close to the peak level stage (~1000 μm in leaf length) of tooth growth were selected for this analysis. One stage point is at the earlier stage, at 700–800 μm in leaf length, and the other is at the later stage, at 1200–1300 μm in leaf length. The results showed that in both Col-0 and *wox1*, the size of cells in the tooth gradually increases during the phase between these two stage points (Fig. 1G). The cell size in the tooth region of *wox1*, excluding the elongated margin cells, was comparable with that in Col-0 at both stage points (Fig. 1G, H). However, the cell size at the flanking region was dramatically reduced in *wox1* compared with that in Col-0 at both stage points (Fig. 1G, I). These results suggest that WOX1 negatively regulates leaf serration size, at least in part, by promoting the cell expansion at the sinus.

### CUC3 mediates WOX1 spatio-temporal suppression of tooth development

**Fig. 2.**
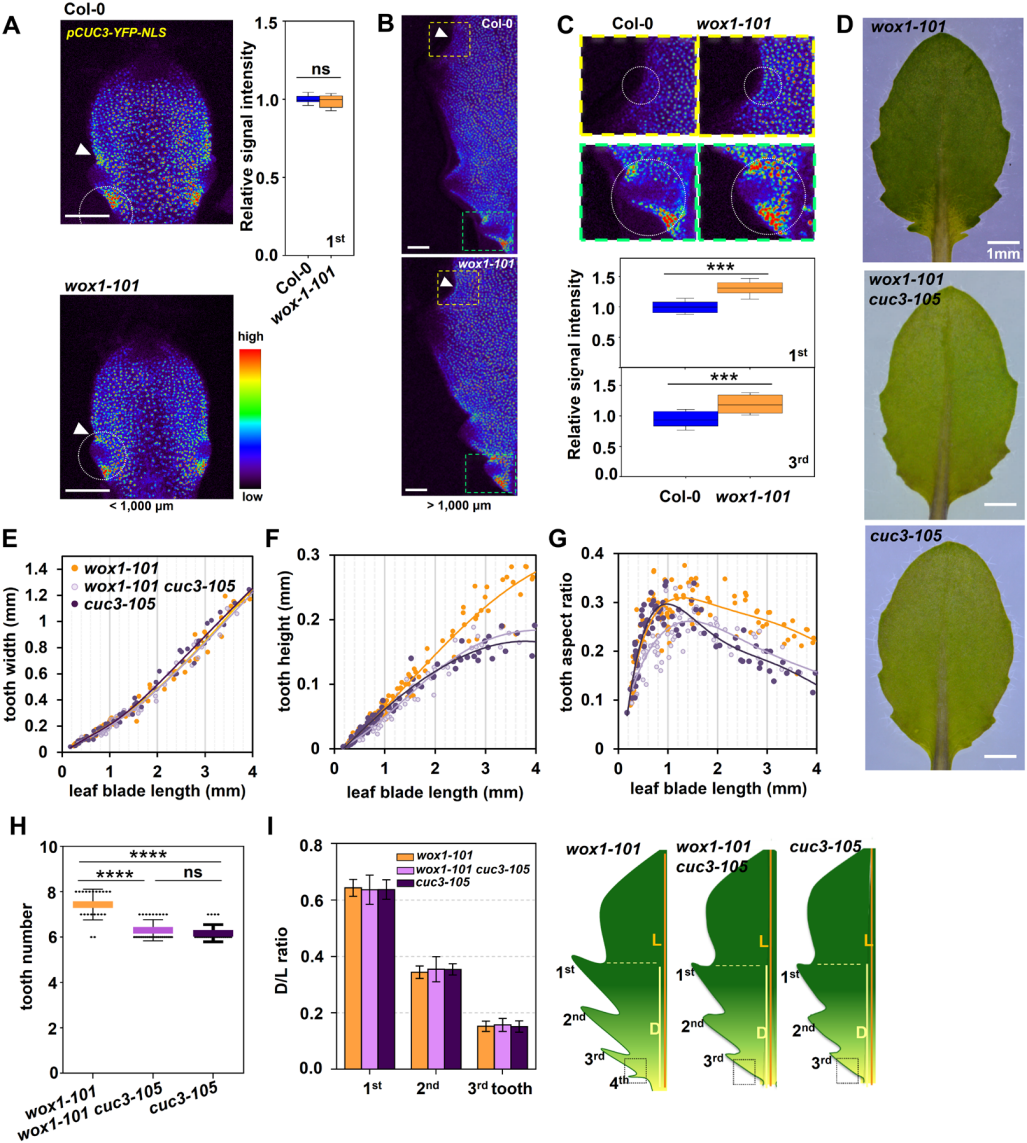
CUC3 mediates negative regulation of tooth development by WOX1. (A) Confocal micrographs of the fifth leaf primordia of Col-0 and *wox1-101* showing the YFP signal from *pCUC3*:*YFP-NLS* expression when the leaf blade length (<1000 μm) corresponds to the earlier stage of development of the first tooth. *CUC3-YFP-NLS* is mainly expressed at the tooth sinus region (white arrowhead). White dashed circles in (A) enclose the first tooth used for the YFP fluorescence intensity measurements in Col-0 (*n*=10) and *wox1-101* (*n*=10) presented in a histogram in the right hand side of (A). ns, non-significant. Scale bars, 80 μm. (B and C) *Z*-projected confocal images of YFP signal in the fifth leaves of Col-0 and *wox1-101* with *pCUC3*:*YFP-NLS* when the leaf length (>1000 μm) corresponds to the later stage of development of the first tooth. The YFP signal intensity in the sinus of teeth 1 or 3 of *wox1-101* (*n*=12) is higher than that in Col-0 (*n*=10) at the same stage. Figures boxed in yellow and green dashed lines in (C) are magnified views from yellow and green boxes in (B), respectively. White arrowheads in (B) indicate the sinus region of the first tooth. White dashed circles in (C) enclose the first tooth sinus region and the third tooth used for the relative YFP fluorescence intensity measurements presented in a histogram at the bottom panel of (C). Scale bars, 50 μm. Student’s *t*-test was performed for statistical analysis. ***P<0.001. (D) The fifth leaves of *wox1-101*, *wox1-101 cuc3-105*, and *cuc3-105* mutants. (E–G) Quantitative analysis of tooth width (E), tooth height (F), and tooth aspect ratio (G) in the first tooth of leaf 5 in *wox1-101* (*n*=220), *wox1-101 cuc3-105* (*n*=144), and *cuc3-105* (*n*=162) mutants following leaf growth. (H) The number of teeth in leaf 5 of *wox1-101* (*n*=20), *wox1-101 cuc3-105* (*n*=30), and *cuc3-105* (*n*=24) mutants. The leaf in *wox1-101* possesses 7–8 teeth while the leaf in *wox1-101 cuc3-105* possesses ~6, which is the same as that in *cuc3-105*. One-way ANOVA was performed within each class, ****P<0.0001. ns, not-significant. (I) Proximal–distal position of the distal sinus of teeth 1–3 relative to leaf blade length in *wox1-101* (*n*=10), *wox1-101 cuc3-105* (*n*=10), and *cuc3-105* (*n*=10) mutants (left panel) and the scheme of leaf 5 in these mutants (right panel). The black dotted square marks the placement of the fourth tooth in *wox1-101* which is inhibited in the leaf of the *wox1-101 cuc3-105* double mutant. D, distance from the distal tooth sinus to the leaf base; L, leaf blade length from tip to the leaf base.

CUCs are crucial for both tooth initiation and outgrowth (Kierzkowski *et al*., 2019; Serra and Perrot-Rechenmann, 2020). Up-regulation of CUCs may account for the excessive tooth growth phenotype (both tooth number and size) in *wox1*. To test this hypothesis, we monitored *CUC2* and *CUC3* expression patterns in Col-0 and *wox1* utilizing *pCUC2:CUC2-YFP* and *pCUC3:YFP-NLS*, respectively. The expression of *CUC2* was comparable in Col-0 and *wox1* (Supplementary Fig. S3A). *CUC3* in Col-0 was mainly expressed at the flanking region of the tooth, which is consistent with previous reports (Hasson *et al*., 2011; Serra and Perrot-Rechenmann, 2020) (Fig. 2A). The global signal intensity of *pCUC3-YFP* in the sinuses along the entire leaf margin was gradually decreased and restricted to the proximal tooth-flanking region as the leaf tooth grew (Fig. 2A, B). *wox1* showed similar dynamic changes of the *pCUC3* activity pattern to that in Col-0, and the YFP signal intensity in *wox1* and Col-0 at the early stage of growth of the first tooth (<1000 μm in leaf blade length) was comparable (Fig. 2A). However, the global YFP signal in the flanking regions of teeth along the whole leaf margin in *wox1* becomes stronger when the leaf blade length reached 1000 μm or longer (up to 1300 μm in leaf length, corresponding to the late stage of the first tooth and the early stage of the third tooth; the point of transition of the third tooth between the early and late stage corresponds to ~2500 μm in leaf length) compared with that in Col-0 at the same stage (Fig. 2B, C; Supplementary Fig. S1I). This led to higher *pCUC3* signal intensity in *wox1*, both in the tooth developed first during the late stage and in the subsequently developed tooth at the early stage (Fig. 2A–C).

It is reported that the induction of CUC3 activity triggers an increase in serration size, whereas inactivation of CUC3 results in the smoothing of leaf margins (Serra and Perrot-Rechenmann, 2020). To investigate whether the enhanced serration size in *wox1* is dependent on elevated *CUC3* expression, *cuc3* loss-of-function mutants were crossed with *wox1*. The enhanced growth in tooth height observed in *wox1* from the late stage of the first and second teeth and from the early stage of the third tooth was suppressed in *wox1 cuc3* (Fig. 2D–G; Supplementary Fig. S4A, B). Additionally, the increased tooth number phenotype in *wox1* was also suppressed due to the lack of CUC3 function: the initiation of the fourth tooth of leaf 5 in *wox1* was inhibited in the *wox1 cuc3* mutant, indicating that the excessive serration number in *wox1* also relies on CUC3 (Fig. 2H, I). These results indicate that CUC3 is involved in WOX1 negative regulation of the first two teeth from the late stage and subsequently developed teeth from the early/initiation stage, suggesting that WOX1 limits tooth outgrowth and number, at least in part, via temporal inhibition of CUC3.

### BZR1 regulates tooth development through CUC3 at the early stage of tooth development

**Fig. 3.**
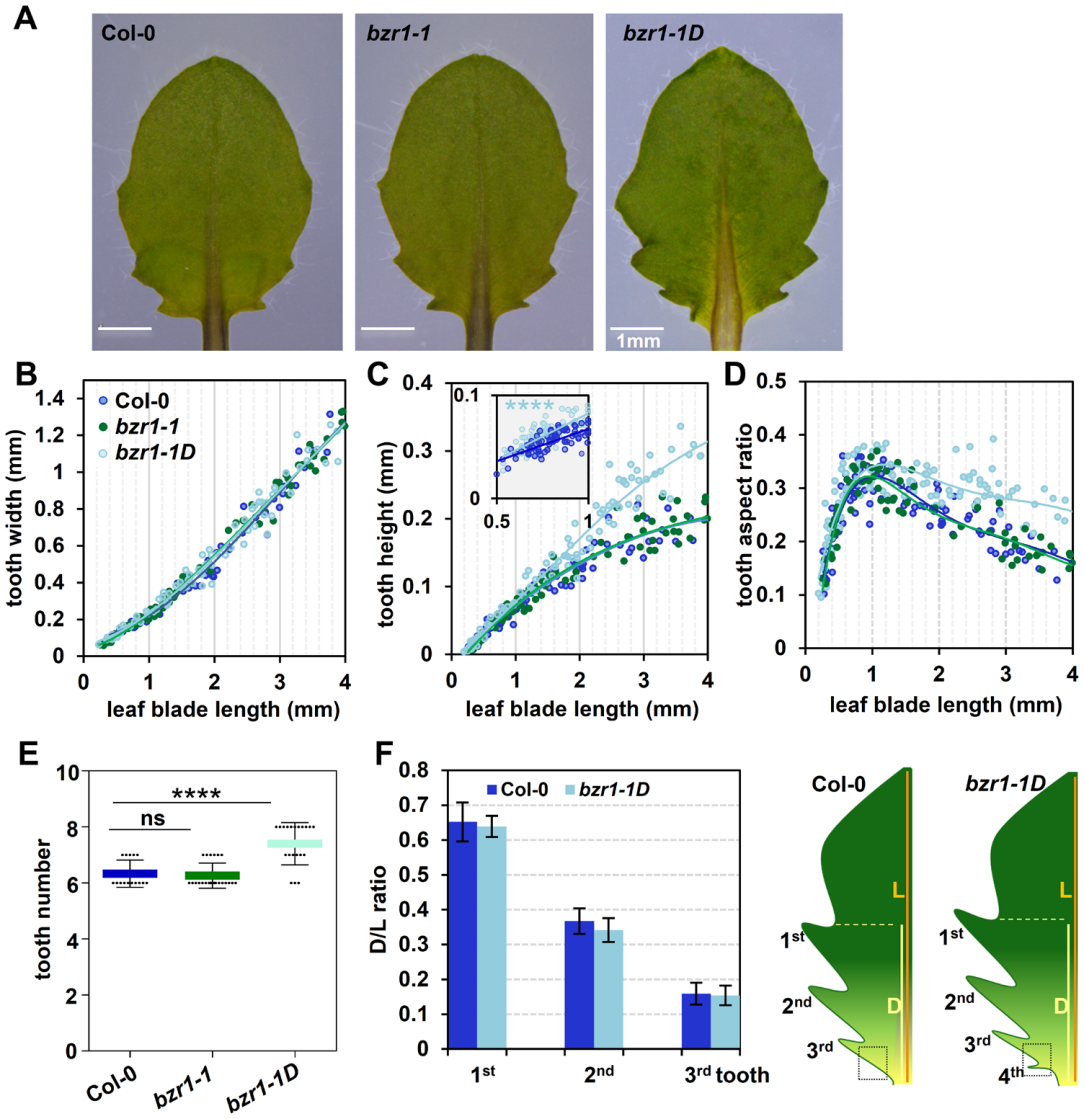
*bzr1-1D* shows enhancement of tooth growth from the early stage as well as increased tooth number. (A) The fifth leaves of Col-0, *bzr1-1*, and *bzr1-1D* mutants. (B–D) Quantitative analysis of tooth width (B), tooth height (C), and tooth aspect ratio (D) in the first tooth of leaf 5 in Col-0 (*n*=181), *bzr1-1* (*n*=178), and *bzr1-1D* (*n*=227) mutants correlated with leaf length increase. The likelihood ratio test was adopted for statistical analysis between Col-0 and *bzr1-1D* on tooth height at the early stage of development of the first tooth. ****P=1.96e-11. (E) The number of teeth in leaf 5 of Col-0 (*n*=15), *bzr1-1* (*n*=23), and *bzr1-1D* (*n*=20) mutants. The leaf in Col-0 possesses ~6 teeth, which is the same as that in *bzr1-1*, while the leaf in *bzr1-1D* possess 1–2 extra teeth compared with Col-0. One-way ANOVA was performed within each class, ****P<0.0001. ns, non-significant. (F) Proximal–distal position of the distal sinus of teeth 1–3 relative to leaf blade length in Col-0 (*n*=10) and *bzr1-1D* (*n*=10) (left panel) and the scheme of leaf 5 in Col-0 and *bzr1-1D* (right panel). The black dotted square marks the placement of the fourth tooth in *bzr1-1D*. D, distance from the distal tooth sinus to the leaf base; L, leaf blade length from tip to the leaf base.

A previous report demonstrated that BZR1 regulates organ boundary formation through modulating both *CUC2* and *CUC3* expression by direct binding to their promoters (Gendron *et al*., 2012). To investigate whether a similar genetic module is also involved in the tooth developmental context, we first examined the role of BZR1 in tooth initiation/growth. Two mutants, *bzr1-1* (loss-of-function mutant) and *bzr1-1D* (gain-of-function mutant), were analyzed. We found that *bzr1-1* exhibited tooth development comparable with Col-0 (Fig. 3A–E; Supplementary Fig. S4C). However, *bzr1-1D* displayed greater tooth protrusion due to increased tooth height without alterting tooth width, compared with Col-0 at the same developmental stage (Fig. 3A–C). The height of the first and second teeth in *bzr1-1D* was increased from the early stage although the timing of initiation remained unaltered (Fig. 3C; Supplementary Figs S2, S4C). This differential growth was sustained at the late stage during tooth development, as the decrease in the tooth aspect ratio in *bzr1-1D* was less than that in the wild type (Fig. 3D; Supplementary Fig. S4C). Furthermore, *bzr1-1D* exhibited an increased number of teeth (1–2 more teeth) compared with Col-0 in leaf 5, although the placement of the first three pairs of teeth remained unaltered (Fig. 3E, F). These results indicate that BZR1 is crucial for tooth outgrowth and may have a potential role in promoting initiation of the fourth tooth.

**Fig. 4.**
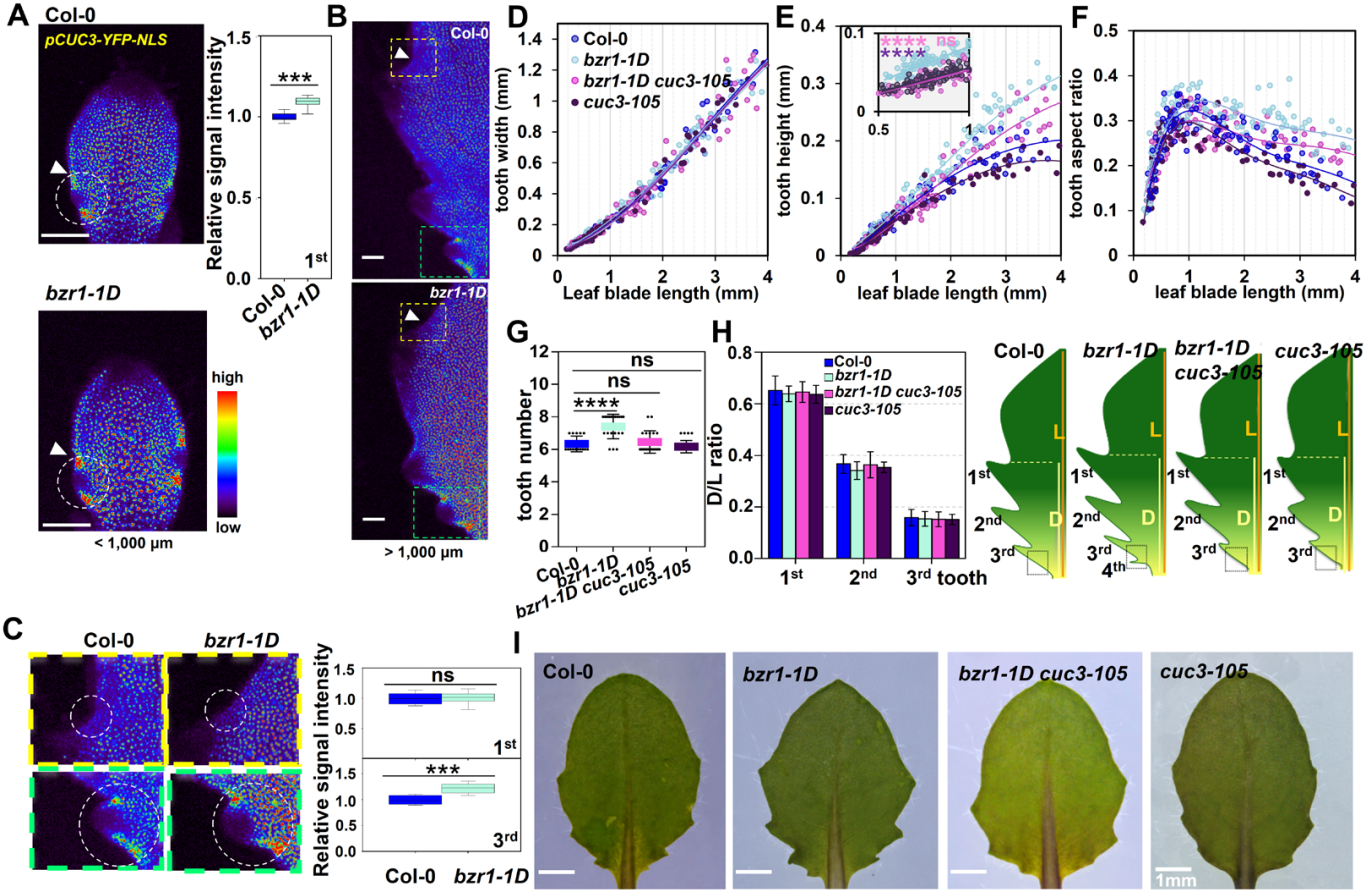
The enhanced serration size at the early stage of tooth development and increased tooth number in *bzr1-1D* depend on CUC3. (A) Confocal micrographs of YFP signal in the fifth leaf primordia at the earlier stage of development of the first tooth of Col-0 and *bzr1-1D* with *pCUC3*:*YFP-NLS* (leaf length <1000 μm). White dashed circles in (A) enclose the first tooth used for the YFP fluorescence intensity measurements in Col-0 (*n*=10) and *bzr1-1D* (*n*=10) presented in a histogram on the right-hand side of (A). YFP signal is mainly detected at the tooth sinus region (white arrowheads). ***P<0.001. Scale bars, 80 μm. (B and C) *Z*-projected confocal images of YFP signal in the fifth leaves of Col-0 and *bzr1-1D* with *pCUC3*:*YFP-NLS* expression when leaf length (>1000 μm) corresponds to the later stage of development of the first tooth. The YFP signal intensity in the sinus of tooth 3 of *bzr1-1D* is higher than that in Col-0, while the signal in the sinus of tooth 1 of *bzr1-1D* was comparable with that in Col-0 at the same stage. Figures boxed in yellow and green dashed lines in (C) are magnified views from yellow and greens boxes in (B), respectively. White dashed circles in (C) enclose the first tooth sinus region and the third tooth used for the relative YFP fluorescence intensity measurements presented in a histogram on the right-hand side of (C). White arrowheads indicate the sinus region of the first tooth. Scale bars, 50 μm. Student’s *t*-test was performed for statistical analysis. ***P<0.001. ns, non-significant. (D–F) Quantitative analysis of tooth width (D), tooth height (E), and tooth aspect ratio (F) in the first tooth of leaf 5 in Col-0 (*n*=181), *bzr1-1D* (*n*=227), *bzr1-1D cuc3-105* (*n*=192), and *cuc3-105* (*n*=162) mutants correlated with leaf length increase. The likelihood ratio test was adopted for statistical analysis between different phenotypes on tooth height at the early stage of development of the first tooth. **** in purple (between *bzr1-1D* and *cuc3-105*), *P*=7.60e -40, ****in pink (between *bzr1-1D* and *bzr1-1D cuc3-105*), *P*=7.90e-38. ns (between *cuc3-105* and *bzr1-1D cuc3-105*), non-significant. (G) The number of teeth in leaf 5 of Col-0 (*n*=15), *bzr1-1D* (*n*=20), *bzr1-1D cuc3-105* (*n*=20), and *cuc3-105* (*n*=24) mutants. The leaf in *bzr1-1D* possesses 7–8 teeth while the leaf in *bzr1-1D cuc3-105* possesses ~6, which is the same as that in *cuc3-105*. One-way ANOVA was performed within each class, ****P<0.0001. ns, non-significant. (H) Proximal–distal position of the distal sinus of teeth 1–3 relative to leaf blade length in Col-0 (*n*=10), *bzr1-1D* (*n*=10), *bzr1-1D cuc3-105* (n=10), and *cuc3-105* (*n*=10) mutants (left panel) and the scheme of leaf 5 in these ecotypes (right panel). The black dotted square marks the placement of the fourth tooth in *bzr1-1D* which is inhibited in the leaf of the *bzr1-1D cuc3-105* mutant. D, distance from the distal tooth sinus to the leaf base; L, leaf blade length from tip to the leaf base. (I) The fifth rosette leaves of Col-0, *bzr1-1D*, *bzr1-1D cuc3-105*, and *cuc3-105* mutants.

The expression of *CUCs* was subsequently investigated during tooth development in *bzr1-1D*. The *CUC2* expression patterns of Col-0 and *bzr1-1D* in the earlier stage of tooth development were comparable. In addition, the CUC2 level was reduced in *bzr1-1D* as the leaf grew, compared with that in Col-0 at the same stage (Supplementary Fig. S3A). The *pCUC3* marker showed a higher expression level in *bzr1-1D* than that in Col-0 at the early stage of tooth development, both in the first tooth and in the subsequently developed tooth (the third tooth) (Fig. 4A–C). This result suggests that elevated CUC3 may underlie abnormal tooth growth in *bzr1-1D* at the early stage of tooth development. In line with this hypothesis, we found no significant difference in tooth height and width between *cuc3* and *bzr1-1D cuc3* during the early stage of tooth growth (Fig. 4D–F; Supplementary Fig. S4D). Furthermore, the increased tooth number in *bzr1-1D* was restored to a normal level in *bzr1-1D cuc3*, as the fourth tooth initiation in leaf 5 of *bzr1-1D* was inhibited in the absence of *CUC3* (Fig. 4G, H). These observations suggest that enhanced expression of *CUC3* induces abnormal tooth development phenotypes in *bzr1-1D* during the early/initiation stage of tooth development.

Although the absence of CUC3 completely blocked the pronounced protrusion phenotype in *bzr1-1D* during the early stage of development of the first and second teeth, the teeth of *bzr1-1D cuc3* at the late stage exhibited an intermediate growth phenotype compared with *bzr1-1D* and *cuc3* single mutants (Fig. 4E, F, I; Supplementary Fig. S4D). In addition, the CUC3–YFP signal intensity was comparable in the first tooth sinus between *bzr1-1D* and Col-0 when the leaf exceeded a length of 1000 μm (corresponding to the late stage of the first tooth) (Fig. 4B, C). These findings suggest that CUC3 and BZR1 may cooperate in parallel to regulate tooth development at the late stage.

### BZR1 is partially involved in WOX1 spatio-temporal inhibition of tooth development

**Fig. 5.**
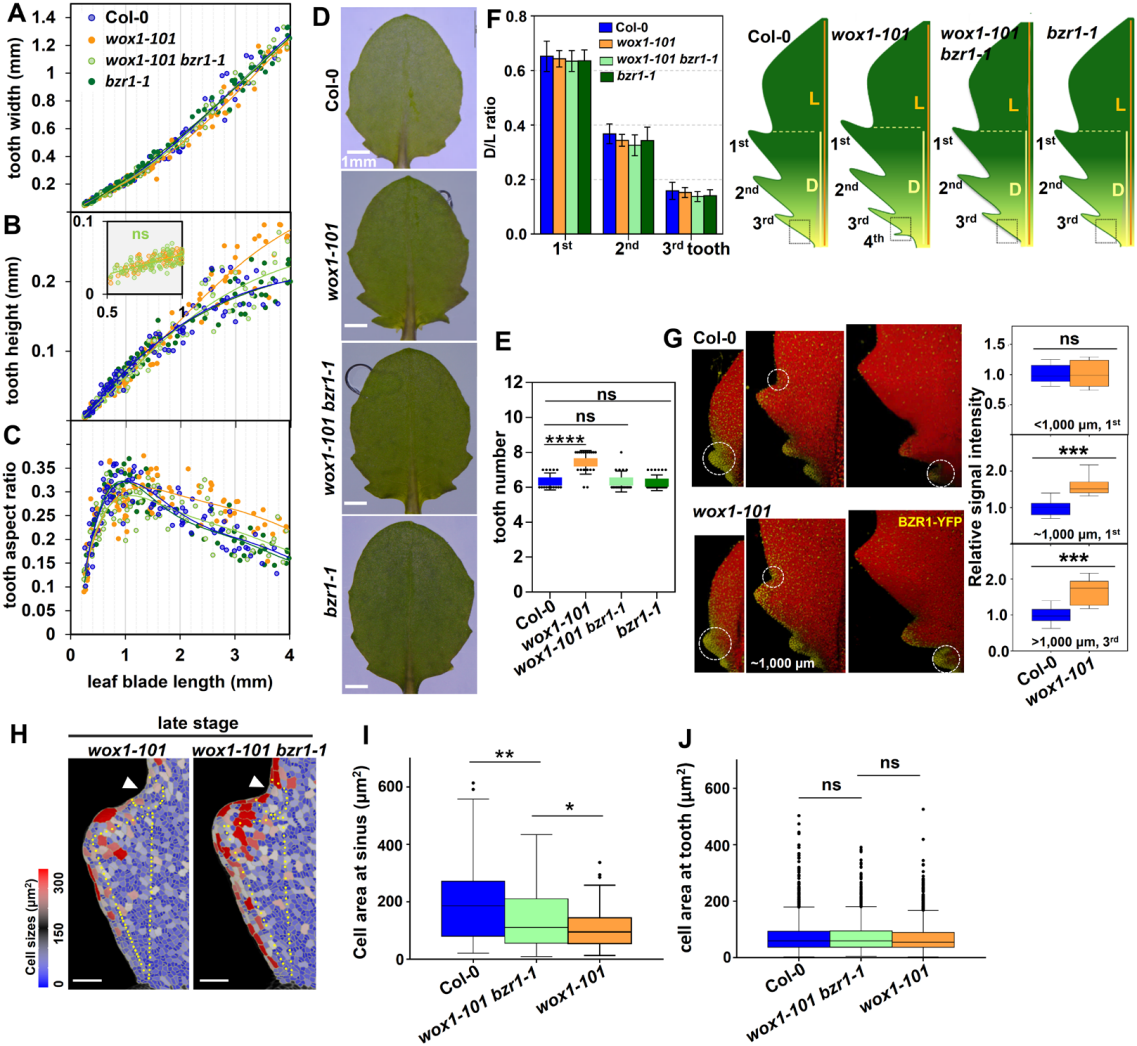
BZR1 partially mediates tooth developmental regulation by WOX1. (A–C) Quantitative analysis of tooth width (A), tooth height (B), and tooth aspect ratio (C) in the first tooth of leaf 5 in Col-0 (*n*=181) *bzr1-1* (*n*=178), *wox1-101* (*n*=220), and *wox1-101 bzr1-1* (*n*=180) mutants correlated with leaf length increase. The likelihood ratio test was adopted for statistical analysis between *wox1-101* and *wox1-101 bzr1-1* of tooth height at the early stage of development of the first tooth. ns, non-significant. (D) The fifth rosette leaves of Col-0, *bzr1-1*, *wox1-101*, and *wox1-101 bzr1-1* mutants. (E) The number of teeth in leaf 5 of Col-0 (*n*=15), *wox1-101* (*n*=20), *wox1-101 bzr1-1* (*n*=18), and *bzr1-1* (*n*=23) mutants. The leaf in *wox1-101* possesses 7–8 teeth while the leaf in *wox1-101 bzr1-1* possesses ~6, which is the same as that in *bzr1-1*. One-way ANOVA was performed within each class, ****P<0.0001. ns, non-significant. (F) Proximal–distal position of the distal sinus of teeth 1–3 relative to leaf blade length in Col-0 (*n*=10), *wox1-101* (n=10), *wox1-101 bzr1-1* (*n*=10), and *bzr1-1* (*n*=10) mutants (left panel) and the scheme of leaf 5 in these ecotypes (right panel). The black dotted square marks the placement of the fourth tooth in *wox1-101* which is inhibited in the leaf of the *wox1-101 bzr1-1* mutant. D, distance from the distal tooth sinus to the leaf base; L, leaf blade length from tip to the leaf base. (G) Three-dimensional confocal micrographs of YFP signal in the fifth leaves of Col-0 and *wox1-101* with *pBZR1:BZR1-YFP* when leaf length is <1000 μm (left; early stage of the first tooth), at ~1000 μm (middle; transition timing of the first tooth), and >1000 μm (right; late stage of the first and early stage of the third tooth). White dashed circles in (G) show the tooth or sinus used for the YFP fluorescence intensity measurements in Col-0 (*n*=10) and *wox1-101* (*n*=10) presented in the histogram on the right-hand side of (G). ***P<0.001. ns, non-significant. (H–J) Heatmaps of cell area in the first tooth of leaf 5 in *wox1-101* and *wox1-101 bzr1-1* at the late stage point (~1200–1300 μm) of tooth development (H), the quantification of cell area at the sinus (I), and at the tooth excluding the elongated margin cells (J). A total of 89, 147, and 193 cells in Col-0 (*n*=7), *wox1-101 bzr1-1* (*n*=8), and *wox1-101* (*n*=9), respectively, were used for the cell area analysis at the tooth sinus in (I). A total of 1711, 1357, and 1464 cells in Col-0 (*n*=7), *wox1-101 bzr1-1* (*n*=8), and *wox1-101* (*n*=9), respectively, were used for cell area analysis at the tooth region (J). Yellow dots in (H) at the sinus region encircle the cells (including yellow dotted cells) used for cell area quantitative analysis in (I). Cells inside the yellow triangles at the tooth region in (H) are used for cell area quantitative analysis in (J). White arrowheads in (H) indicate tooth sinus regions. Scale bars in (H), 50 μm. One-way ANOVA was performed within each class, *P<0.05. **P<0.01. ns, non-significant.

The above results showed that both WOX1 and BZR1, at least partially, control leaf serration via modulation of *CUC3* expression, implying a regulatory interplay between WOX1 and BZR1 during tooth development. To elucidate the genetic relationship between WOX1 and BZR1, *wox1* was crossed with the loss-of-function mutant *bzr1-1.* In the early stage, the fifth leaves of *wox1* and *wox1 bzr1-1* exhibited comparable tooth height with identical width in the first and second teeth (Fig. 5A, B; Supplementary Fig. S4E). However, the enlarged protrusion observed in the first two teeth at the late stage of *wox1* compared with that in Col-0 was partially restored in *wox1 bzr1-1* (Fig. 5B, C; Supplementary Fig. S4E). In addition, the *wox1*-mediated enhancement of size of the third tooth at the early stage was dramatically reduced in this double mutant (Supplementary Figs S1H, S4F). Furthermore, the tooth number abnormality was rescued in *wox1 bzr1-1*, where the fourth tooth formation in leaf 5 of *wox1* was suppressed in the *wox1 bzr1-1* mutant (Fig. 5D–F). These findings suggest that BZR1 participates in WOX1 regulation of tooth development from the late stage of the first developed teeth, and from the early/initiation stage of the subsequently developed teeth.

To investigate whether WOX1 inhibits tooth development through the regulation of temporal expression patterns of *BZR1*, we compared the *BZR1* expression pattern in *wox1* and Col-0 by observing the YFP signal of *pBZR1:BZR1-YFP*. In Col-0, BZR1–YFP was broadly distributed across the leaf blade, with a higher signal intensity in the marginal tooth region before the leaf blade reached ~1000 μm (peak level stage of the first tooth) (Fig. 5G; Supplementary Fig. S5A). A basipetal progression of reduction in BZR1–YFP signal was observed as the leaf grew, and the difference in BZR1–YFP signal intensity between the leaf blade and marginal tooth region was noticeable in the newly formed teeth at the leaf base when the leaf blade exceeded 1000 μm (Fig. 5G; Supplementary Fig. S5A). BZR1–YFP in *wox1* displayed similar expression pattern/level changes to those in Col-0 during the early stage of growth of the first tooth. However, a decrease of BZR1–YFP signal in the leaf margin towards the leaf base was retarded in *wox1* when the leaf blade reached ~1000 μm (Fig. 5G; Supplementary Fig. S5A). This resulted in a higher BZR1–YFP signal intensity in *wox1* in the first and subsequently developed teeth from the late and early/initiation stage, respectively. These results suggest that WOX1 temporally inhibits *BZR1* expression to restrict tooth outgrowth and tooth number. Additionally, in line with the observation that *bzr1-1D* exhibits a higher CUC3 signal at the early stage of tooth growth, the elevated CUC3 signal in the third tooth of *wox1* at the early stage was reduced to a level comparable with that in Col-0 in the absence of BZR1 (Figs 2C, 4C; Supplementary Fig. S5B, C). The *wox1 bzr1* double mutant maintains higher CUC3 activity in the first tooth at the late stage compared with Col-0, similar to the result in *wox1* (Fig. 2B, C; Supplementary Fig. S5B, C). This is consistent with the observation that *bzr1-1D* has no effect on CUC3 activity in the first tooth at the late stage (Fig. 4B, C).

To further investigate the role of BZR1 in the differential tooth growth features observed between Col-0 and *wox1*, we conducted a cell-level comparison of the first tooth among Col-0, *wox1*, and *wox1 bzr1-1* at the later developmental stage point (1200–1300 μm in leaf length). The result showed that the introduction of *bzr1-1* in *wox1* led to a partial release of cell expansion inhibition at the tooth sinus (Fig. 5H, I), while the cell area in the tooth region excluding elongated margin cells remained unaltered (Fig. 5H, J). Collectively, these results support the hypothesis that BZR1, at least partially, mediates WOX1 suppression of tooth overgrowth in the flanking region via inhibition of cell expansion.

## Discussion

**Fig. 6.**
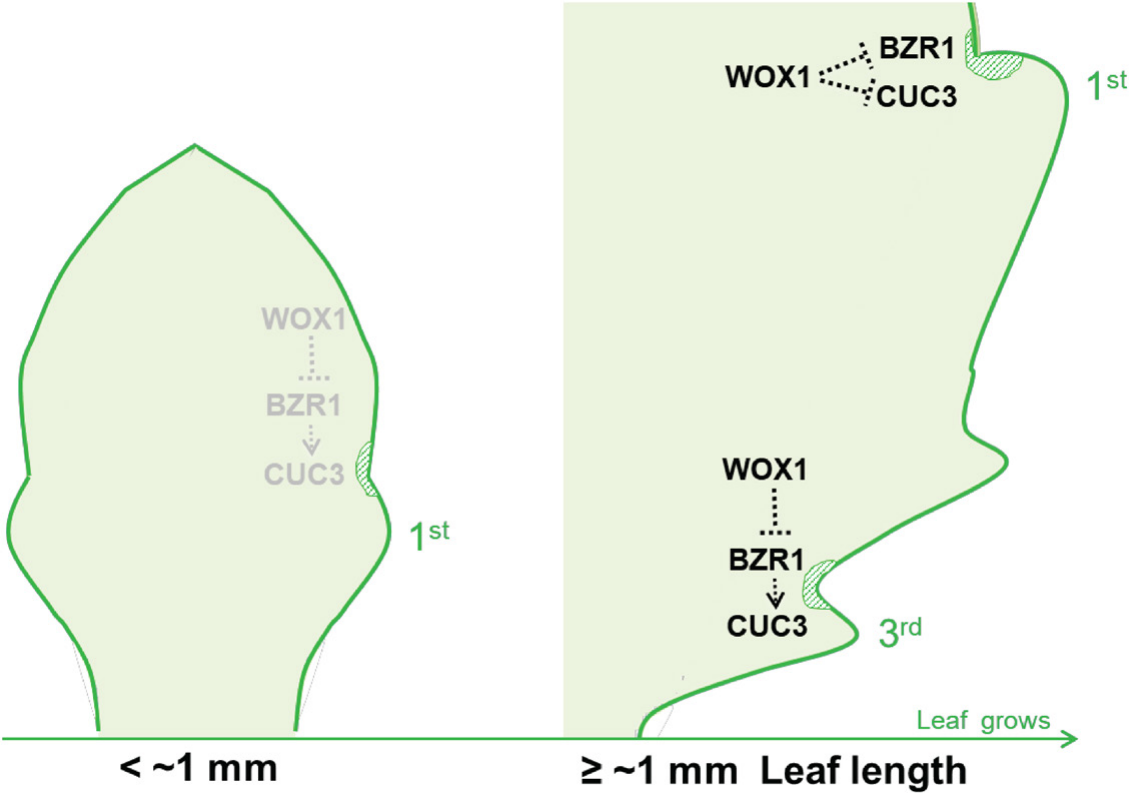
A conceptual diagram summarizing WOX1 negative regulation of tooth development via temporally limiting BZR1 and CUC3 expression. WOX1 does not affect BZR1 and CUC3 expression until the leaf grows to ≥1 mm. It limits BZR1 and CUC3 expression in parallel from the late stage of tooth development in the first two teeth (represented by the first tooth in the diagram), while it restricts CUC3 activity in a BZR1-dependent manner from the early/initiation stage in the subsequently developed tooth in leaf 5 (represented by the third tooth in the diagram). The green hatched areas indicate the tooth sinus region.

Our results suggest that WOX1 prevents excessive tooth development to maintain a moderate level of leaf protrusion size and number, by temporally suppressing the expression of *CUC3* and *BZR1*. The regulation of *CUC3* and *BZR1* expression by WOX1 is related to leaf development, as the inhibition does not occur until the leaf is longer than 1 mm (Fig. 6). Furthermore, we demonstrate that BZR1 mediates WOX1 limitation on *CUC3* expression at the early/initiation stage of tooth development. Consequently, WOX1 prevents excessive *CUC3* and *BZR1* expression in the first teeth to be developed (the first and second) from the late stage of tooth development, while limits *CUC3* expression in the subsequently developed teeth (the third and fourth) from the early/initiation stage which is mediated by BZR1 (Fig. 6).

The cellular level analysis of the first tooth reveals that WOX1 prevents excessive tooth outgrowth at the late stage by promoting cell expansion. WOX1 and PRS have primarily been reported to promote leaf marginal meristematic activity (Nakata *et al*., 2012; Alvarez *et al*., 2016). We found that although WOX1 limits excessive tooth development when the leaf is longer than 1 mm, it is required for the emergence of the first tooth, which occurs at a stage when the leaf length is <1 mm (~250 μm in leaf length) (Nikovics *et al*., 2006) (Supplementary Figs S2, S6). WOX1 and PRS work redundantly to promote *CUC2* expression that is indispensable for serration formation (Supplementary Fig. S6). This is consistent with previous research showing that ectopic expression of *WOX1* elevates the level of *CUC2* (Nakata *et al*., 2018). Serration formation results from the establishment and maintenance of a new tooth growth axis. It seems to be a common function of WOXs in controlling the growth axis of organs. For instance, knockdown of SlLAM1 in tomato, an ortholog of WOXs, led to a reduced number of secondary leaflets (Wang *et al*., 2021). In summary, WOX1 seems to perform a proliferative function to promote tooth formation at the earlier stage, and functionally changes to promoting cell expansion at the later stage to prevent excessive tooth development. This study uncovers a role for WOX1 in regulating cell growth, and the differential regulation by WOX1 of cell growth at different stages relies on the developmental context.

The temporal regulation of *BZR1* and *CUC3* by WOX1 may account for the mechanism behind WOX1 regulation of cell expansion, as both of these genes are involved in the regulation of cell expansion at the tooth sinus (Serra and Perrot-Rechenmann, 2020) (Fig. 5I) However, WOX1 temporal regulation of *BZR1* and *CUC3* is unlikely to be solely dependent on the overlap of their expression pattern, since WOX1 is expressed along the leaf margin, and *CUC3* and *BZR1* expression largely overlaps with the expression of *WOX1* at the early stage of the first tooth (Nakata *et al*., 2012; Alvarez *et al*., 2016) (Figs 2A, 5G; Supplementary Fig. S5D). These observations suggest that other unknown factors are likely to be involved in WOX1 function in tooth development. NGATHA (NGA) and CINCINNATA (CIN-TCP) transcription factors may be considered as potential candidates for the following reasons. (i) TCPs regulate the spatio-temporal expression of *WOX1* and *PRS* along the leaf margin during leaf primordia growth. In the absence of TCPs and NGAs, WOX1 and PRS fail to functionally transition into regulating cell expansion, even in older leaves. They still maintain the proliferative activity that leads to continuous cell proliferation at the margins of all lateral organs (Alvarez *et al*., 2016). (ii) Some NGAs and TCPs are expressed distally at the tip of young leaf primordia, and are gradually extended to the leaf serrations or confined to the proximal region of older leaves (Alvarez *et al*., 2016). (iii) CsWOX1 is reported to interact with CsTCP4a in cucumber (Wang *et al*., 2020). (iv) *tcp* mutants exhibit increased leaf complexity, while plants overexpressing mTCP2/3/4 (resistant to MIR319) display smooth leaf margins (Palatnik *et al*., 2003; Koyama *et al*., 2007; Schommer *et al*., 2014). Thus, TCPs are potential candidates to cooperate with WOX1 to enable its role in cell expansion regulation during tooth development, and the WOX1-mediated spatio-temporal inhibition of tooth development may be a consequence of the expression pattern of TCPs.

Besides its role on restricting tooth size, WOX1 also inhibits initiation of the fourth tooth. The fifth leaf in Col-0 possesses three pairs of teeth, while *wox1* possesses four, with the placement of the first three pairs of teeth remaining unaltered. CUC2 is responsible for tooth initiation, and the *cuc2* mutant exhibits a smooth leaf margin (Nikovics *et al*., 2006; Kawamura *et al*., 2010). We found that increased *CUC2* expression leads to enhanced tooth size that is consistent with previous research (Larue *et al*., 2009), whereas the tooth number in *cuc2-1D* (gain-of-function) was comparable with that in Col-0. This indicates that CUC2 has no contribution to initiation of the fourth tooth in leaf 5 (only the primary tooth is the focus of this study) (Supplementary Fig. S3B, C). CUC3 was reported to be involved in promoting early tooth outgrowth, although the *cuc3* mutant still possesses teeth along the margin (Hasson *et al*., 2011; Maugarny-Cales *et al*., 2019). Here we found that the absence of CUC3 leads to a decrease in tooth size, which is consistent with previous studies, and the tooth number remains unaffected (first to third pair of teeth in leaf 5) (Fig. 2F–H). Furthermore, the increase in tooth number in *wox1* is correlated with an elevated *CUC3* level and is recovered to a normal level when crossed with *cuc3*, indicating that CUC3 may also be involved in regulation of tooth initiation. A detailed analysis showed that CUC3 is involved in initiation of the fourth tooth in *wox1* (Fig. 2H, I). This indicates the potential existence of distinct regulatory modules governing the initiation of the first three teeth and of the fourth tooth. In accordance, CUC3 activity is elevated in *wox1* while *CUC2* expression remains unaffected (Fig. 2B, C; Supplementary Fig. S3A). The differential regulation of CUC2 and CUC3 by WOX1 suggests the existence of other factor(s) that preferentially acts on the control of the *CUC3* expression. We identify BZR1 as a factor that enhances *CUC3* expression during the early/initiation stage of tooth development, although CUC3 seems not to be involved in the regulation of BZR1 at the late stage of tooth growth (Fig. 4E, F; Supplementary Fig. S4D). In summary, WOX1 inhibits the initiation of the fourth tooth in Col-0 by restricting CUC3 activity mediated by BZR1, thereby limiting excessive tooth production.

Previous reports indicate that enhanced *BZR1* expression caused organ fusion phenotypes such as fused stamens and cotyledons via directly suppressing the expression of *CUC2* and *CUC3* (Gendron *et al*., 2012). In this work, BZR1 was found to regulate early tooth development through up-regulating CUC3, suggesting that the regulation on CUC3 by BZR1 might be developmental context dependent. There are examples of developmental context-dependent regulations for other transcription factors. For instance, ethylene response factor 3 (ERF3), an AP2/ERF transcription factor protein in rice, was reported to promote the expression of the cytokinin-responsive gene *RR2* during crown root initiation, while repressing *RR2* during crown root elongation (Zhao *et al*., 2015). BZR1 may serve as both an activator and repressor of *CUC* genes depending on the developmental context. Consistently, while BZR1 is regarded as a positive regulator of BR signaling to promote organ growth, it is crucial to note that BZR1 plays a dual role BR homeostasis and growth responses. In *Arabidopsis*, although *bzr1-1D* (gain-of-function) increases cell elongation in darkness to promote hypocotyl growth, it exhibits a dwarf phenotype when grown under light (He *et al*., 2005). EjBZR1 in loquat has also been reported to be a repressor of organ growth, specifically in suppressing fruit enlargement by limiting cell expansion (Su *et al*., 2021). Therefore, the role of BZR1 in plant development could be both positive and negative, depending on the developmental context. BZR1 switches cellular programs via activating and repressing different target genes. It recruits additional factors such as Groucho/TUP1-like transcriptional co-repressor TOPLESS (TPL) to enable its role in repressing target genes (Oh *et al*., 2014).

Unraveling the downstream pathways governed by *WOX1*–BZR1–CUC3 will deepen our understanding of the regulatory mechanisms behind spatio-temporal regulation to achieve proper development of leaf serration. Cytokinin signaling is also involved in inhibiting cell expansion at the tooth sinus, and a mutant with altered cytokinin homeostasis exhibits smooth leaf margins in *Arabidopsis* (Hajheidari *et al*., 2019; Navarro-Cartagena and Micol, 2023). Meanwhile, *BZR1*, *CUC3*, and the *WOX1* ortholog gene *STF* (in *Medicago truncatula*) were reported to act upstream/downstream of cytokinin signaling to perform their functions in various developmental contexts (Li *et al*., 2010; Tadege *et al*., 2011; Zu *et al*., 2022). All this information implies an interconnection between cytokinin signaling and WOX1–BZR1–CUC3 in tooth developmental regulation, which will be the subject of future studies.

## Supplementary data

The following supplementary data are available at *JXB* online.

Fig. S1. Lack of WOX1 but not PRS leads to leaf tooth developmental abnormality.

Fig. S2. The timing of tooth initiation in Col-0, *wox1-101*, and *bzr1-1D*.

Fig. S3. CUC2 does not contribute to the excessive tooth development phenotype in *wox1-101* and *bzr1-1D* compared with that in Col-0.

Fig. S4. The second/third tooth growth features in leaf 5 among different mutants.

Fig. S5. Confocal micrographs of YFP or GFP signal in the fifth leaf primordia of Col-0 and/or mutants.

Fig. S6. WOX1 and PRS redundantly promote *CUC2* expression and initiation of the first tooth.

Table S1. Primers used in this study.

erae443_suppl_Supplementary_Figures_S1-S6_Table_S1

## Data Availability

All data reported in this paper will be shared by the corresponding authors upon request.
