# Supplementary material for: WOX1 controls leaf serration development via temporally restricting BRASSINAZOLE RESISTANT 1 and CUP SHAPED COTYLEDON 3 expression in Arabidopsis
Source: J Exp Bot. 2024 Oct 30;76(2):478–92. doi: 10.1093/jxb/erae443 (PMC11714755; doi:10.1093/jxb/erae443)
Supplement: erae443_suppl_Supplementary_Figures_S1-S6_Table_S1 [file erae443_suppl_supplementary_figures_s1-s6_table_s1.pdf]

Supplementary Figures and legends

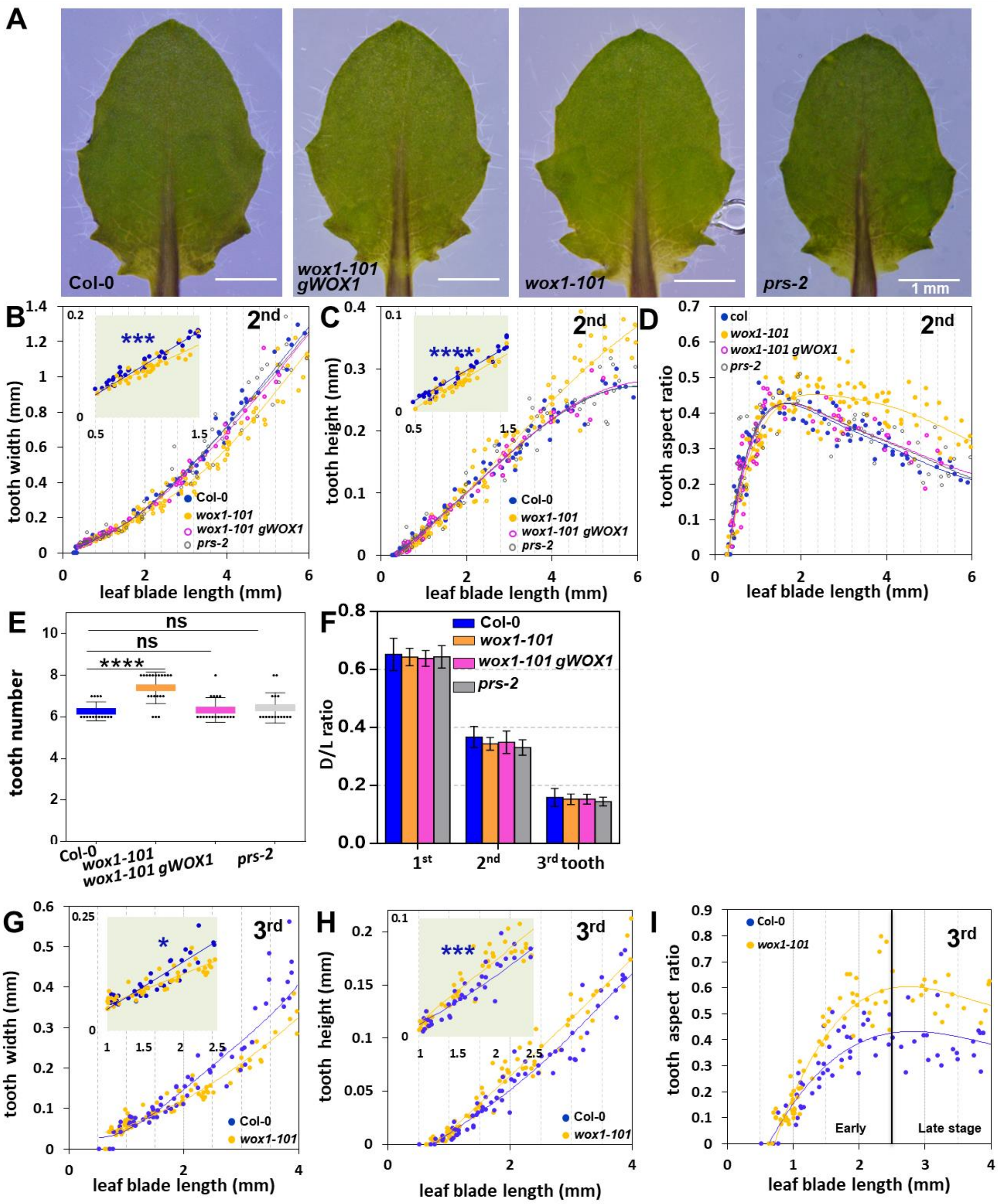

Supplementary Fig. S1. Lack of WOX1 but not PRS leads to leaf tooth developmental abnormity.

(A) The fifth leaves in Col-0, *prs-2*, *wox1-101* and *pWOX1*:*WOX1*; *wox1-101*. (B-D) Quantitative analysis of tooth width (B), tooth height (C) and tooth aspect ratio (D) in the 2nd tooth of leaf 5 in Col-0, *prs-2*, *wox1-101* and *pWOX1*:*WOX1*; *wox1-101* following leaf growth. Likelihood-ratio test was adopted for statistical analysis between Col and *wox1-101* on tooth width and height at the early stage of the 2nd tooth development. *** in (B), $p=0.00046$, **** in (C), $p=4.52e-08$. (E) The tooth number in leaf 5 of Col-0, *wox1-101*, *wox1-101 gWOX1* and *prs-2* mutants. The leaf

in *wox1-101* possesses 7-8 teeth while the leaf tooth number in *wox1-101 gWOX1* recovered to normal level, which is the same with that in Col-0 and *prs-2*. One-way ANOVA analysis was performed within each class, ****, P<0.0001. ns, no significance. (F) Relative proximal-distal position of the distal sinus of tooth 1 to 3 against leaf blade length in Col-0, *wox1-101*, *wox1-101 gWOX1* and *prs-2*, as shown in (Biot *et al*., 2016). D: Distance from the distal tooth sinus to the leaf base. L: Leaf blade length from tip to the leaf base. (G-I) Quantitative analysis of tooth width (G), tooth height (H) and tooth aspect ratio (**I**) in the 3rd tooth of leaf 5 in Col-0 and *wox1-101* following leaf growth. Likelihood-ratio test was adopted for statistical analysis between Col and *wox1-101* on tooth width and height at the early stage of the 3rd tooth development. * in (G), p=0.0498, *** in (H), p=0.000167. The black vertical line in (I) separates the early and the late stage of the 3rd tooth growth.

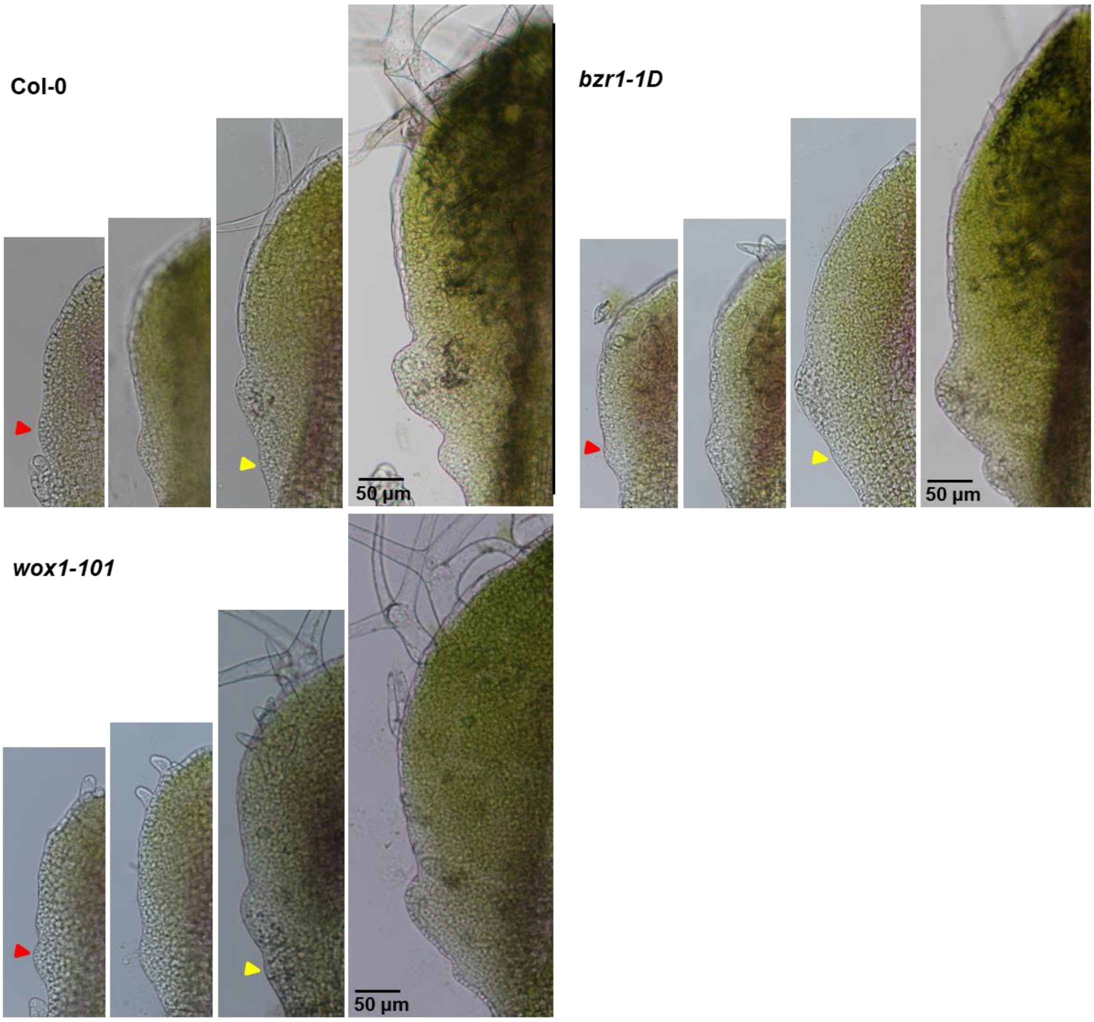

Supplementary Fig. S2. The timing of tooth initiation in Col-0, *wox1-101* and *bzr1-1D*. Developmental sequences of the 1st and the 2nd teeth in leaf 5 of Col-0, *wox1-101* and *bzr1-1D* (n⩾9 for each ecotype at each stage). Red and yellow arrowheads indicate the 1st and 2nd teeth initiation along the leaf margin, respectively. Tooth initiation timing was comparable among them.

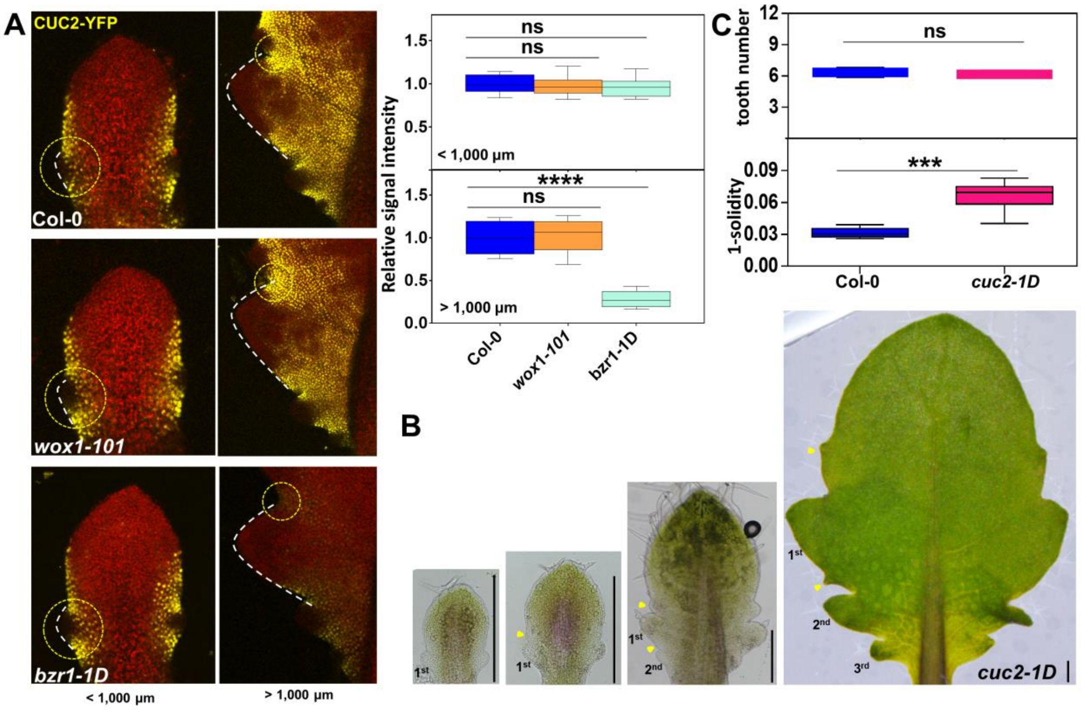

Supplementary Fig. S3. CUC2 does not contribute to the excessive tooth development phenotype in *wox1-101* and *bzr1-1D* compared to that in Col-0.

(A) Z-projected confocal micrographs of the fifth leaves showing YFP signal from *pCUC2:CUC2-YFP* when leaf length corresponds to the earlier (figures in left panel) and the later (figures in right panel) stage of the 1st tooth development. Yellow dashed circles in (A) circled the 1st tooth or tooth sinus used for the YFP fluorescence intensity measurements in Col-0 (n=8) and *wox1-101* (n=8) presented in histogram in the right hand side of (A). White dashed lines indicate the tooth outline. ****, P<0.0001. ns, no significance. (B) The progression of successive tooth development as leaf grows in gain-of-function mutant *cuc2-1D* (n⩾7 for each stage). Yellow arrowheads indicate the intercalary tooth referred from (Kierzkowski *et al.*, 2019). (C) Tooth number (up panel) and tooth growth level (bottom panel) in the fifth leaves of Col-0 (n=10) and *cuc2-1D* (n=11). *cuc2-1D* showed enhanced tooth size while comparable tooth number compared to that in Col-0 (only primary tooth was considered in this study). ***, P<0.001. ns, no significance.

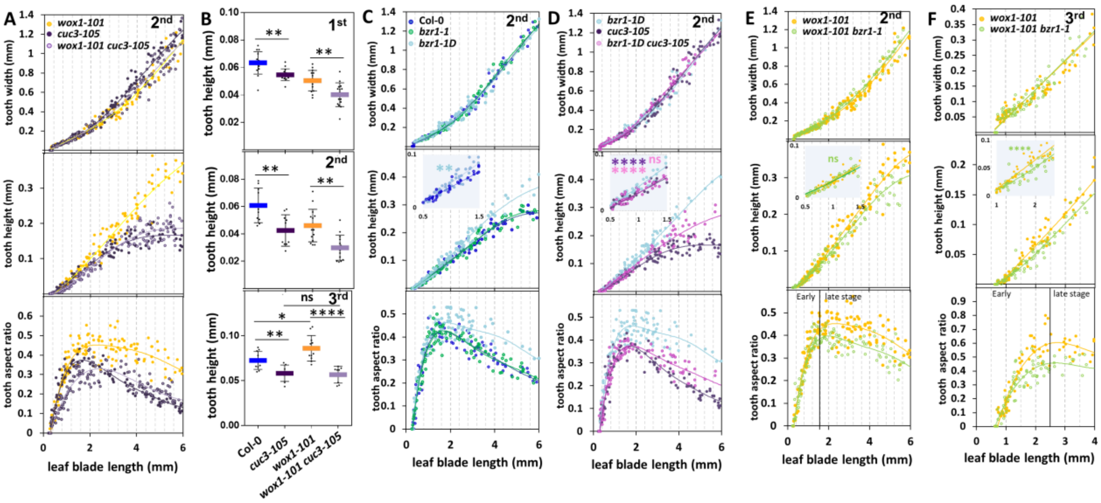

Supplementary Fig. S4. The 2nd/3rd tooth growth features in leaf 5 among different mutants.

(A) Quantitative analysis of tooth width, tooth height and tooth aspect ratio in the 2nd tooth of leaf 5 in Col-0, *wox1-101*, *cuc3-105* and *wox1-101 cuc3-105* plants correlated with leaf length increase. (B) Statistical analysis on tooth height between Col-0 and *cuc3-105*, *wox1-101* and *wox1-101* cuc3-105 at the early stage of the 1st (0.8-1 mm in leaf length), 2nd (1.3-15 mm in leaf length) and 3rd (2.3-2.5 mm in leaf length) tooth. Student's *t*-test was performed for statistical analysis. Consistent with the previous result, *cuc3-105* showed reduced tooth height than that in Col-0 at the early stage of 1st-3rd tooth growth (Maugarny-Cales *et al*., 2019). Loss of *CUC3* in *wox1-101* background also leads to reduced tooth height in the 1st-3rd tooth of *wox1-101*, and the differential growth of tooth height between *wox1-101* and *wox1-101 cuc3-105* is comparable to that between Col-0 and *cuc3-105* in the 1st and 2nd. Although *wox1-101* showed increased tooth height at the early stage of the 3rd tooth than that in Col-0, loss of *CUC3* in Col and *wox1-101* background leads to similar tooth height. *, P<0.05.**, P<0.01. ****, P<0.0001. ns, no significant difference. (C-F) Quantitative analysis of tooth width, tooth height and tooth aspect ratio in the 2nd/3rd tooth of leaf 5 in Col-0, *bzr1-1*, *bzr1-1D*, *bzr1-1D cuc3-105*, *cuc3-105*, *wox1-101* and *wox1-101 bzr1-1* plants correlated with leaf length increase. Likelihood-ratio test was adopted for statistical analysis between different phenotypes on tooth height at the early stage of the 2nd/3rd tooth development in (C-F). (C) **, significant difference between Col-0 and *bzr1-1D*, p=0.0015. (D)**** in purple color (between *bzr1-1D* and *cuc3-105*), p=1.32e-08, **** in pink color (between *bzr1-1D* and *bzr1-1D cuc3-105*), p= 7.40e-10. ns (between *cuc3-105* and *bzr1-1D cuc3-105*), no significance. (E) ns, no significant difference between *wox1-101* and *wox1-101 bzr1-1* at the early stage of the 2nd tooth development. (F) ****, significant difference between *wox1-101* and *wox1-101 bzr1-1* at the early stage of the 3rd tooth development, p=1.23e-06.

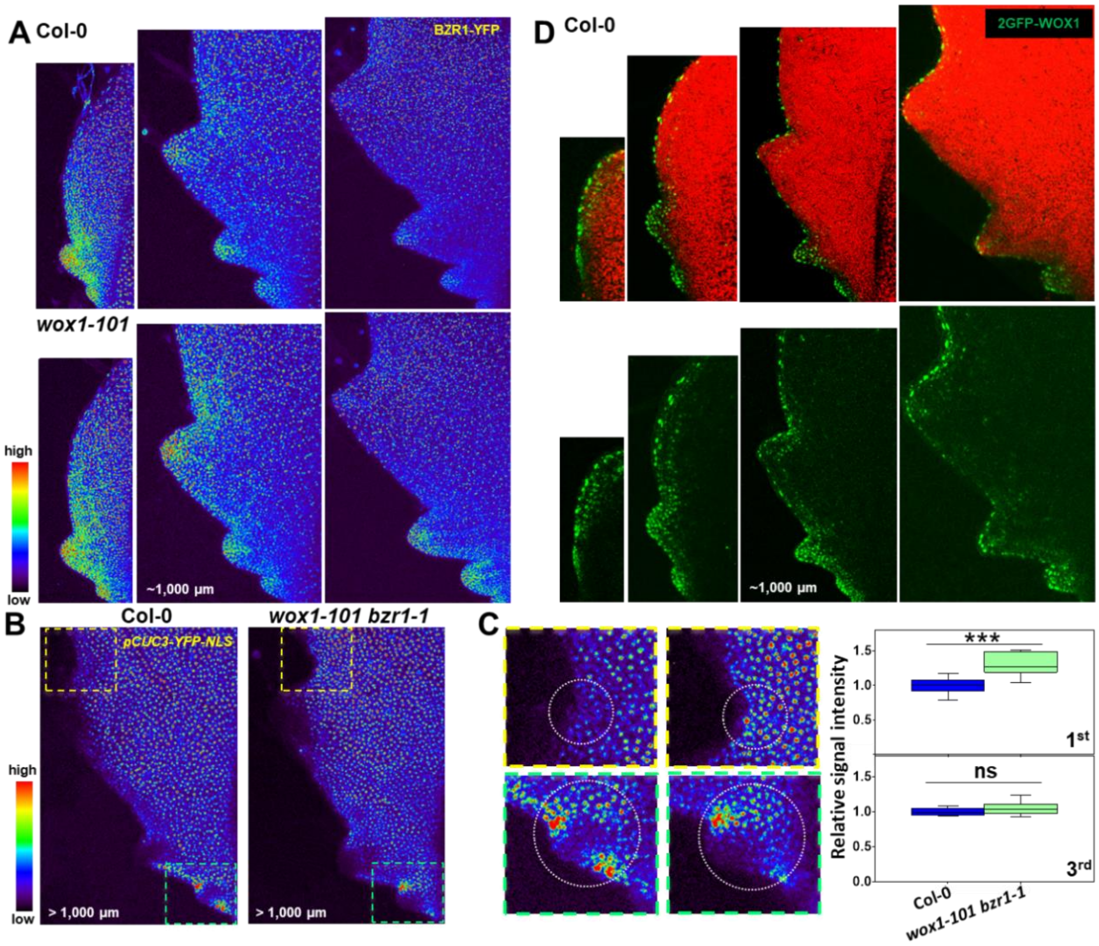

Supplementary Fig. S5. Confocal micrographs of YFP or GFP signal in the fifth leaf primordia of Col-0 and/or mutants.

(A) Z-projected confocal micrographs of YFP signal in the fifth leaves of Col-0 and *wox1-101* with *pBZR1:BZR1-YFP*, related to Figure 5G. (B-C) Z-projected confocal images of YFP signal in the fifth leaves of Col-0 and *wox1-101 bzr1-1* with *pCUC3:YFP-NLS* when leaf length (>1,000 µm) corresponds to the later stage of the 1st tooth development. Figures boxed in yellow and green dashed lines in (C) are magnified view from yellow and green boxes in (B), respectively. White dashed circles in (C) circled the 1st tooth sinus region and the 3rd tooth used for the relative YFP fluorescence intensity measurements presented in histogram at the right hand side of (C). Student's *t*-test was performed for statistical analysis. ***, $P < 0.001$. ns, no significant difference. (D) Z-projected confocal images of GFP signal in the fifth leaves of Col-0 with *pWOX1:2GFP-WOX1* when leaf length corresponds to the earlier, transition timing (~1,000 µm in leaf blade length) and later stage of the first tooth development.

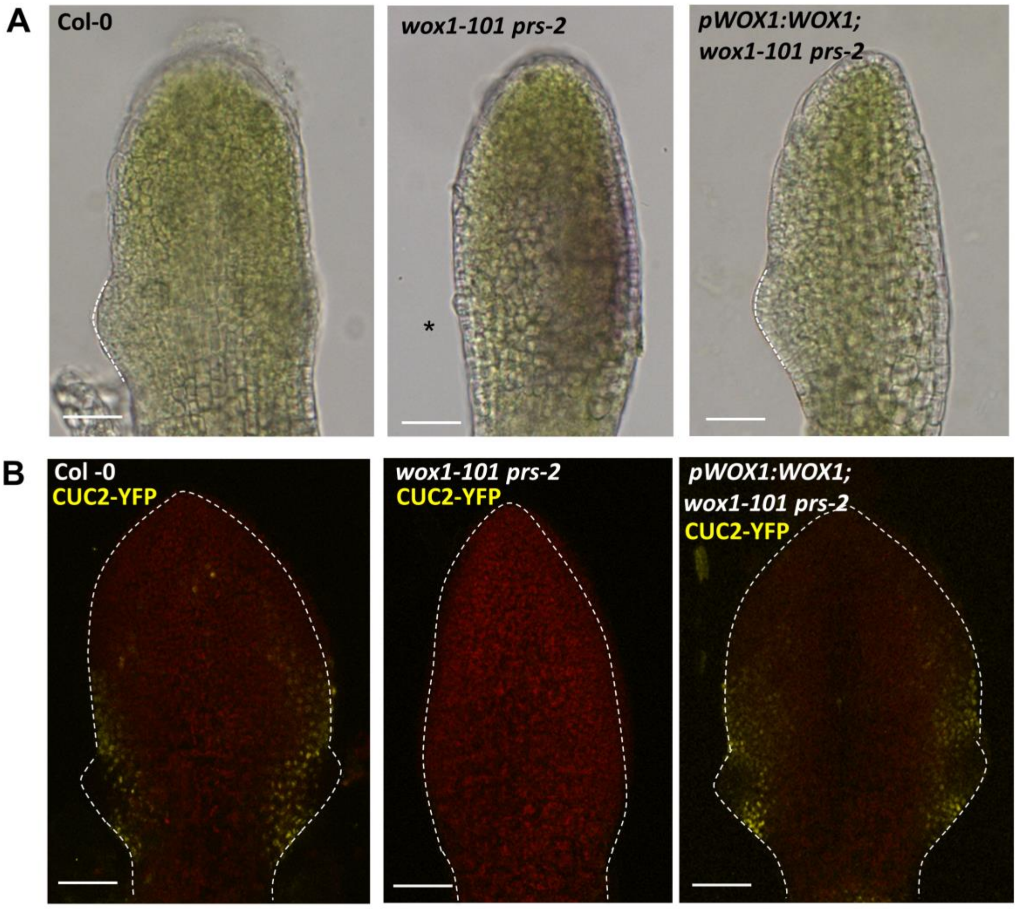

Supplementary Fig. S6. WOX1 and PRS redundantly promote CUC2 expression and the 1st tooth initiation.

(A) The third leaf primordia of Col-0, *wox1-101 prs-2* and *pWOX1*:*WOX1*; *wox1-101 prs-2* plants. White dotted line indicates the tooth outline, * indicate the smooth margin of leaf primordia with the failure of tooth formation. (B) Confocal micrographs of YFP signal in the third leaf primordia of Col-0, *wox1-101 prs-2* and *pWOX1*:*WOX1*; *wox1-101 prs-2* plants with *pCUC2*:*CUC2-YFP*. White dashed lines indicate the leaf margin with tooth outline. Scale bars in (A) and (B), 20 µm.

Supplementary Table S1 Primers information used in this study

Primer name	Primer sequence (5'-3')	Notes
pBZR1-F	GCCTAATTCATCGAACCCTC	
BZR1-R	ACCACGAGCCTTCCCATTTC	
pCUC2-F	ACGACGGCCAGTGCCAAGCTTGTGTCCAACATT CAAGACCG	
pCUC2-R	AAACTTGTTGATAACTCTAGATAAGAAGAAAGA TCTAAAGCTTTTG	
gCUC2-F	ATGGACATTCCGTATTACCAC	
gCUC2-R	GTAGTTCCAAATACAGTCAAGTCC	
pCUC3-F	CACTGGATACACGAACAGAGT	
pCUC3-R	CTTTTACTTAATATAACTGAAAAAGAG	
WOX-LP	CTTTCCAGTCTCTCTTTCCCTTGTT	Genotyping primers
WOX-RP	AGATATATACCTCTGGTTGCGTGTC	
PRS-LP	ACGTAATGTAATAATTTTGGGAGCA	
PRS-RP	TTTGGTGCAGTAATATTCATTCATC	
CUC3-LP	AGATGTGTTAAGCGAACTCGC	
CUC3-RP	CTGTCCTCCCCATACTAAGCC	
LBb1.3	ATTTTGCCGATTTCGGAAC	
D0065-T	GTTAAATATGAAAATGAAAACGGTAGAGG	
